# Oncotype DX Breast Recurrence Score Distribution and Chemotherapy Benefit Among Women of Different Age Groups With HR-Positive, HER2-Negative, Node-Negative Breast Cancer in the SEER Database

**DOI:** 10.3389/fonc.2020.01583

**Published:** 2020-10-30

**Authors:** Ran Cheng, Xiangyi Kong, Xiangyu Wang, Yi Fang, Jing Wang

**Affiliations:** Department of Breast Surgical Oncology, National Cancer Center/National Clinical Research Center for Cancer/Cancer Hospital, Chinese Academy of Medical Sciences and Peking Union Medical College, Beijing, China

**Keywords:** HR+/HER2–/N0 breast cancer, detailed age grouping, Oncotype DX breast recurrence score, SEER database, chemotherapy benefit

## Abstract

**Objective:** To explore the distribution of Oncotype DX Breast Recurrence Score (RS), the proportion of receiving chemotherapy, and the relationship between RS and chemotherapy benefit according to detailed age groups in women with hormone receptor-positive, human epidermal growth factor receptor 2-negative, node-negative (HR+/HER2−/N0) breast cancer.

**Methods:** This was an extensive, comprehensive, population-based retrospective study. Data on individuals with breast cancer were obtained from the Surveillance, Epidemiology, and End Results (SEER) Program. The cohort was divided into five groups by age (≤ 35, 36–50, 51–65, 66–80, >80 years). RS distribution and chemotherapy proportion among different age groups were analyzed, and the overall survivals between patients receiving chemotherapy and those not/unknown were compared in each age group.

**Results:** The study cohort comprised 49,539 patients and the largest age group was 51–65 years. The percentage of patients with low-risk RS (0–10) increased with age, whereas those with intermediate-risk RS (11–25) decreased with age (except for the group of 36–50 years, which had the highest rate of intermediate-risk RS). The age group ≤35 years has the greatest rate of high-risk RS (26–100). The proportion of receiving chemotherapy decreased with age in all RS risk categories. Overall survival was benefited by chemotherapy only in the age group of 66–80 years with intermediate- and high-risk RS, and chemotherapy seemed to do more harm than good for patients older than 80 years.

**Conclusions:** In the present study, we identified the distribution of RS, the proportion of receiving chemotherapy, and the relationship between RS and chemotherapy benefit according to a detailed age grouping for women with HR+/HER2−/N0 breast cancer, which may help in making individualized clinical decisions.

## Introduction

Breast cancer is the most common malignant tumor in women worldwide (1, 2). Implementation of early screening and self-examination has resulted in increasing numbers of breast cancer patients being diagnosed at an early stage (3). The optimal treatment of early-stage hormone receptor-positive (HR+), human epidermal growth factor receptor 2-negative (HER2–) and lymph node-negative (N0) breast cancer is currently controversial (4, 5). Some patients achieve a higher survival rate with endocrine therapy alone, whereas others require chemotherapy to reduce the recurrence rate and mortality (6). Because breast cancer is heterogeneous, not all patients benefit equally from chemotherapy (7). Meanwhile, the economic burden and adverse reactions caused by chemotherapy increase the patients' economic and psychological pressure and reduce their life quality and compliance (8).

In patients with HR+/HER2– and lymph node-negative breast cancer, the Oncotype recurrence score (Oncotype DX Breast Recurrence Score, RS) assay has been shown to predict the risk of breast cancer recurrence and of gaining benefit from adjuvant chemotherapy (9–13). It is a reverse-transcriptase-polymerase-chain-reaction-based assay that detects the expression levels of 21 different genes in breast cancer tumor tissues (14). The platform contains 16 breast cancer-related genes and 5 reference genes, the interaction between them being identified to determine the characteristics of the breast cancer and predict the recurrence index and chemotherapy benefit (15). According to the Trial Assigning Individualized Options for Treatment (TAILORx) study (9–12), Recurrence Scores (RS) are stratified as low-risk (0–10), intermediate-risk (11–25), and high-risk (26–100). That study found that Oncotype recurrence scores provide the risk of recurrence at 9 years in patients with HR+/HER2–/N0 breast cancer and identified the implications for various groups. They found a very low risk of recurrence on endocrine therapy alone in the low-risk group (RS of 0–10) and a high likelihood of benefit from chemotherapy in the high-risk group (RS of 26–100). However, in patients at intermediate-risk (RS of 11–25), the TAILORx suggested that other clinical factors, such as age and clinical risk, should be considered when advising adjuvant chemotherapy in addition to endocrine therapy. For example, the data suggested that chemotherapy is associated with some benefit in women aged ≤50 years with an RS of 16–25 (9). Clinical risk stratification integrating tumor size and histologic grade could provide additional prognostic information when it is added to RS, enabling identification of premenopausal women who may derive some benefit from more effective therapy than a course of tamoxifen (11).

Notably, in studies investigating RS younger patients are usually underrepresented, which is of concern because younger women tend to have more aggressive forms of the disease (16). Most patients in published studies are older; thus, there are few published data regarding the relationship between chemotherapy benefit and RS in younger women. A recent single academic-center retrospective cohort study revealed that the relationship between pathological features and RS is consistent irrespective of age (17). RS may therefore be useful irrespective of age. However, the limitations of that study included a small sample size (total *n* = 344, younger *n* = 133, and older *n* = 211) and that it was a single institution study. Moreover, only two age groups (age <50 vs. age ≥50) were compared. In the present study, we aimed to obtain a large sample from the Surveillance, Epidemiology and End Results (SEER) database and to divide patients into smaller age groups to enable a more in-depth analysis for the distribution of RS, the proportion of receiving chemotherapy, and the relationship between RS and chemotherapy benefit among women of different age groups with HR+/HER2−/N0 breast cancer.

## Methods

This was a large, population-based retrospective cohort research. Data on women with breast cancer were obtained from the SEER database, which was linked to Oncotype DX Breast Recurrence Score assay results for analyses following SEER approval. Women who had undergone Oncotype DX testing between January 2010 and December 2015 were retrospectively and sequentially identified. The study cohort included patients with ER-positive and/or progesterone receptor (PR)-positive HER2-negative invasive breast cancer as defined by the SEER reported ER and PR immunohistochemistry results. HER2 status was only reliably captured from 2010 and is therefore only available beyond this time. Only patients with no lymph node metastases (N0) were selected for analysis. Oncotype DX Breast Recurrence Score, clinicopathologic information (such as tumor size, histologic grade, PR status, histological type) and other information (such as age at diagnosing, race, and chemotherapy administered record) were extracted from the database. Furthermore, the following exclusion criteria were applied: (1) missing RS data; (2) RS more than 12 months after a breast cancer diagnosis; (3) distant metastases (M1) at initial diagnosis; (4) multiple primary malignant tumors, and (5) incomplete follow-up.

The eligible cohort was divided into five groups by age (≤ 35, 36–50, 51–65, 66–80, >80 years), these cut-offs having been selected on the basis of previously reported studies (9, 17–21). The groups of Oncotype RS were defined as low-risk (RS of 0–10), intermediate-risk (RS of 11–25), and high-risk (RS of 26–100) in accordance with the stratification standard of the TAILORx study (12). The primary survival outcome of this study was overall survival (OS).

To investigate the primary objective, in each age group, the percentages of RS risk categories were calculate, the rates of adjuvant chemotherapy were also assessed within each RS risk category according to the chemotherapy records obtained from SEER database. Kaplan-Meier curves were plotted and compared for overall survival probability using the log-rank tests between patients with chemotherapy records of “Yes” and “No/Unknown.” *P*-values <0.05 were considered to denote statistical significance.

## Results

**Figure 1:**
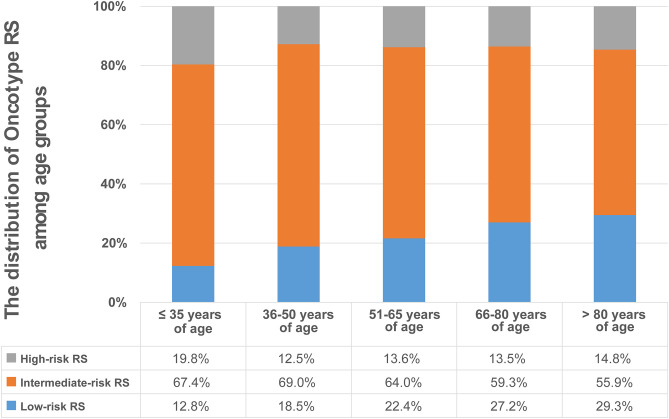
Distribution of Oncotype RS in the five age groups. The percentage of low-risk RS patients increased with age, whereas that of intermediate-risk RS patients decreased with age (except for the group of 36–50 years, which had the highest rate of intermediate-risk RS). The greatest rate of high-risk RS patients was in the group aged ≤ 35 years, followed by the group aged > 80 years, then the 51–65 and 66–80 years old groups, the 36–50 years of age group had the smallest proportion.

The study cohort comprised 49,539 patients who were divided into five age groups. The largest age group was 51–65 years (*n* = 23, 759; 48.0%) followed by 36–50 years (*n* = 12, 474; 25.2%), 66–80 years (*n* = 12, 160; 24.5%), ≤ 35 years (*n* = 672; 1.4%), and over 80 years (*n* = 474; 1.0%). Patient characteristics according to age group are shown in Table 1. Low-, intermediate- and high-risk RS groups comprised 11,164 (22.5%), 31,731 (64.1%), and 6,644 (13.4%) patients, respectively. The percentage of low-risk RS patients increased with age, whereas that of intermediate-risk RS patients decreased with age (except for the group of 36–50 years, which had the highest rate of intermediate-risk RS). The greatest rate of high-risk RS patients was in the group aged ≤ 35 years (19.8%), followed by the group aged > 80 years (14.8%), then the 51–65 and 66–80 years old groups (13.6% and 13.5%, respectively), the 36–50 years of age group having the smallest proportion (12.5%) (Figure 1). The median RS in the ≤ 35, 36–50, 51–65, 66–80, and over 80 years of age groups being 18 (range, 0–65), 16 (range, 0–73), 16 (range, 0–74), 15 (range, 0–69), and 15 (range, 0–55), respectively (Table 1).

**Table 1:** Patient characteristics according to age group.

**Characteristics**	**Overall (*n =* 49,539)**	**≤ 35 years of age (*n =* 672)**	**36–50 years of age (*n =* 12,474)**	**51–65 years of age (*n =* 23,759)**	**66–80 years of age (*n =* 12,160)**	**> 80 years of age (*n =* 474)**
**Age at diagnosing**						
Median (range)	**58 (18–92)**	**32** (18–35)	46 (36–50)	58 (51–65)	70 (66–80)	83 (81–92)
**Race (%)**						
White	**40,413 (81.6)**	486 (72.3)	9650 (77.4)	19,580 (82.4)	10,296 (84.7)	401 (84.6)
Black	**4,008 (8.1)**	84 (12.5)	1,078 (8.6)	1,851 (7.8)	951 (7.8)	44 (9.3)
Others^※^	**4,768 (9.6)**	96 (14.3)	1,645 (13.2)	2,157 (9.1)	843 (6.9)	27 (5.7)
Unknown	**350 (0.7)**	6 (0.9)	101 (0.8)	171 (0.7)	70 (0.6)	2 (0.4)
**RS**						
Mean (±SD)	**16.71 (8.66)**	19.62 (9.38)	16.99 (8.13)	16.84 (8.66)	16.04 (9.05)	15.84 (9.60)
Median (range)	**16 (0–74)**	18 (0–65)	16 (0–73)	16 (0–74)	15 (0–69)	15 (0–55)
**RS risk group (TAILORx standard) (%)**						
Low (0–10)	**11,164 (22.5)**	86 (12.8)	2,309 (18.5)	5,317 (22.4)	3,313 (27.2)	139 (29.3)
Intermediate (11–25)	**31,731 (64.1)**	453 (67.4)	8,602 (69.0)	15,203 (64.0)	7,208 (59.3)	265 (55.9)
High (26–100)	**6,644 (13.4)**	133 (19.8)	1563 (12.5)	3,239 (13.6)	1,639 (13.5)	70 (14.8)
**Histology-broad groupings (%)**						
8500–8549: Ductal and lobular neoplasms	**47,799 (96.5)**	628 (93.5)	1,2053 (96.6)	22,969 (96.7)	11,704 (96.3)	445 (93.9)
Others^#^	**1,740 (3.5)**	44 (6.5)	421 (3.4)	790 (3.3)	456 (3.7)	29 (6.1)
**Tumor size (%)**						
T1	**37,238 (75.2)**	481 (71.6)	9,556 (76.6)	18,153 (76.4)	8,760 (72.0)	288 (60.8)
T2	**11,316 (22.8)**	177 (26.3)	2,696 (21.6)	5,178 (21.8)	3,107 (25.6)	158 (33.3)
T3/T4	**757 (1.5)**	11 (1.6)	174 (1.4)	313 (1.3)	234 (1.9)	25 (5.3)
TX (Unknown)	**228 (0.5)**	3 (0.4)	48 (0.4)	115 (0.5)	59 (0.5)	3 (0.6)
**Histological grades (%)**						
I	**14,562 (29.4)**	155 (23.1)	3,674 (29.5)	7,222 (30.4)	3,398 (27.9)	113 (23.8)
II	**26,416 (53.3)**	344 (51.2)	6,666 (53.4)	12,530 (52.7)	6,613 (54.4)	263 (55.5)
III/IV	**7,564 (15.3)**	159 (23.7)	1,913 (15.3)	3,532 (14.9)	1,874 (15.4)	86 (18.1)
Unknown	**997 (2.0)**	14 (2.1)	221 (1.8)	475 (2.0)	275 (2.3)	12 (2.5)
**Progesterone receptor status (%)**						
Positive	**45,317 (91.5)**	642 (95.5)	12,018 (96.3)	21,435 (90.2)	10,824 (89.0)	398 (84.0)
Negative/Borderline	**4,191 (8.4)**	30 (4.5)	446 (3.6)	2,308 (9.7)	1,331 (10.9)	76 (16.0)
Unknown	**31 (0.1)**	0 (0.0)	10 (0.1)	16 (0.1)	5 (0.1)	0 (0.0)
**Chemotherapy records (%)**						
Yes	**8,698 (17.6)**	276 (41.1)	2,923 (23.4)	4,193 (17.6)	1,286 (10.6)	20 (4.2)
No/Unknown	**4,0841 (82.4)**	396 (58.9)	9,551 (76.6)	19,566 (82.4)	10,874 (89.4)	454 (95.8)

※ *American Indian/AK Native, Asian/Pacific Islander*.

# *8000–8009: Unspecified neoplasms; 8010–8049: Epithelial neoplasms, NOS; 8050–8089: Squamous cell neoplasms; 8140–8389: Adenomas and adenocarcinomas; 8390–8429: Adnexal and skin appendage neoplasms; 8440–8499: Cystic, mucinous and serous neoplasms; 8560–8579: Complex epithelial neoplasms*.

**Figure 2:**
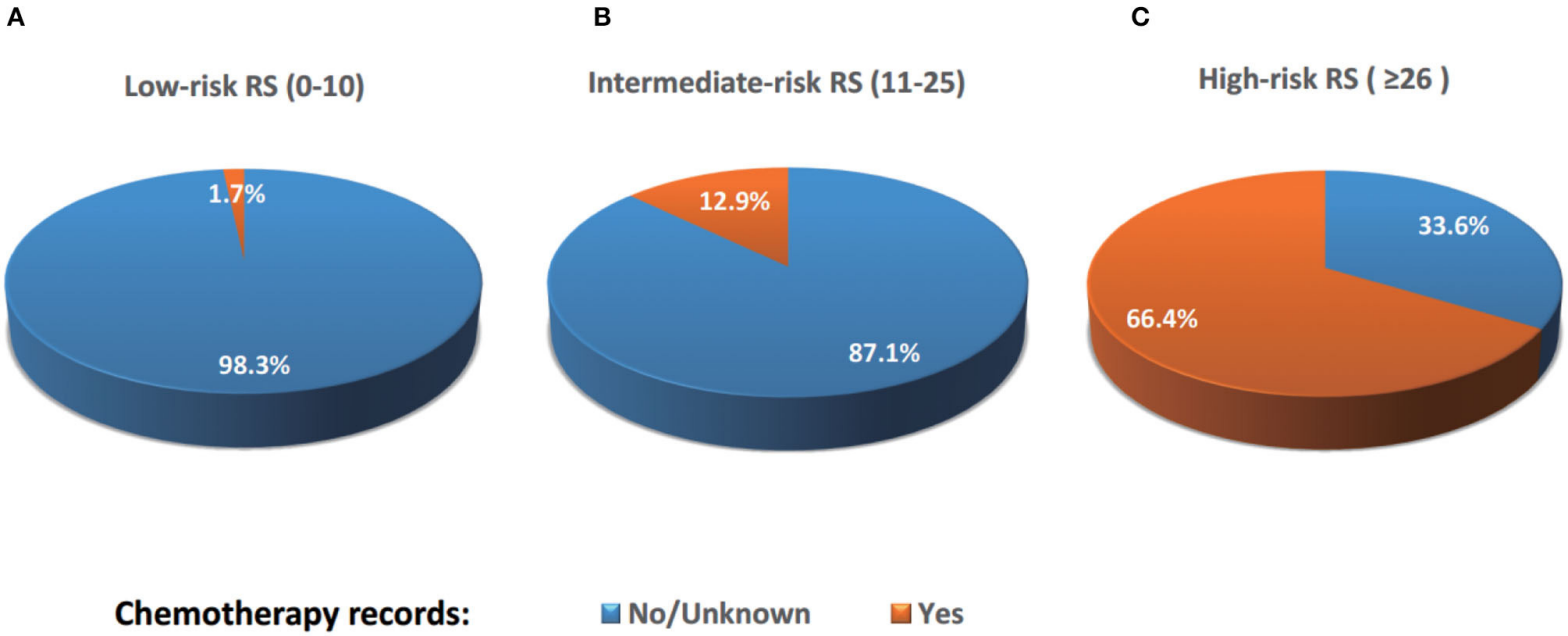
Percentages of patients receiving chemotherapy according to RS risk categories. 1.7%, 12.9%, and 66.4% of patients with low-, intermediate-, and high-risk RS were recorded with “Yes” of chemotherapy, respectively, while 98.3%, 87.1%, and 33.6% of patients with low-, intermediate-, and high-risk RS were recorded with “No/Unknown” of chemotherapy, respectively.

**Figure 3:**
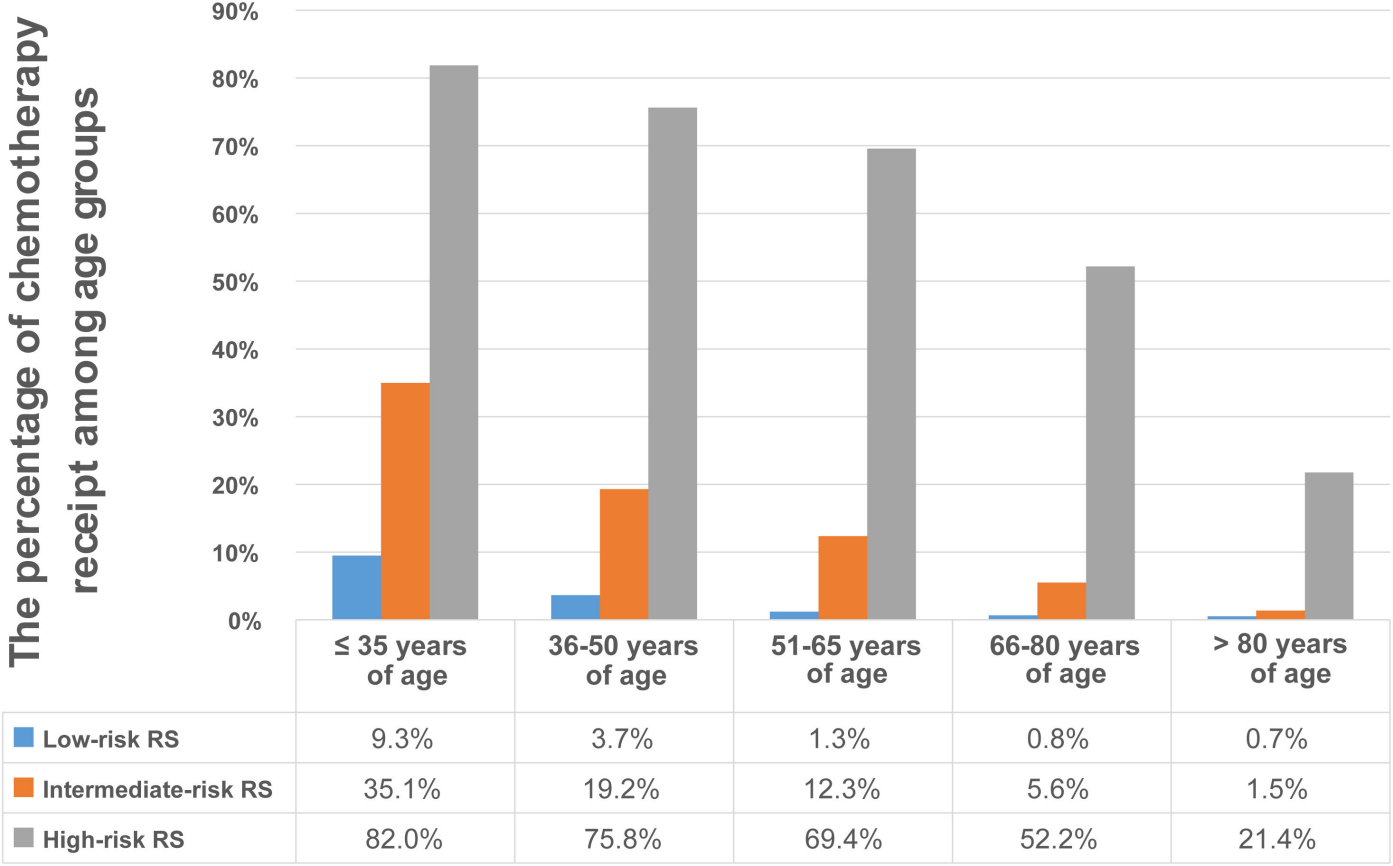
Percentages of patients receiving chemotherapy according to age group. the “Yes” chemotherapy record rates decreased with age in all RS risk categories. Patients aged ≤ 35 years with high-risk RS had the highest rate of receiving chemotherapy (82.0%), whereas those aged over 80 years with low-risk RS had the lowest rate (0.7%).

A total of 8,698 (17.6%) patients were recorded with “Yes” in the chemotherapy records entry (Table 1). The percentage of “Yes” records were related to the results of the Oncotype RS assay (Figure 2). 1.7%, 12.9%, and 66.4% of patients with low-, intermediate-, and high-risk RS were recorded with “Yes,” respectively. The proportion of “Yes” records of chemotherapy in the group aged ≤ 35 years was the highest, while in the group aged > 80 years was the lowest (Table 1). When analyzing the percentage of chemotherapy receipt among low-, intermediate-, and high-risk RS group in five age groups, we found that the “Yes” chemotherapy record rates decreased with age in all RS risk groups. The greatest proportion of women recorded with “Yes” of chemotherapy (82.0%) was in the ≤ 35 years old group with high-risk RS, whereas the smallest proportion (0.7%) was in the over 80 years old group with low-risk RS (Figure 3).

**Figure 4:**
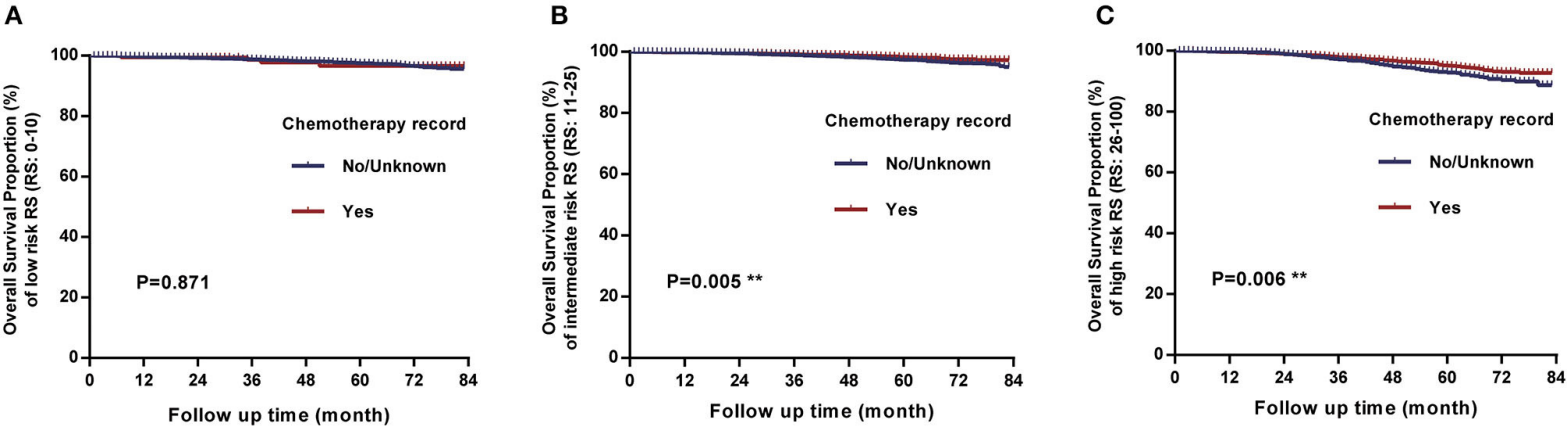
Comparison of overall survival between patients receiving chemotherapy and those not receiving chemotherapy or unknown. **(A)** There was no significant difference between the two treatment groups when RS of 0 to 10 (*P* = 0.871). **(B)** There was a significant difference when RS of 11 to 25 (*P* = 0.005). **(C)** There was a significant difference when RS ≥ 26 (*P* = 0.006).

**Figure 5:**
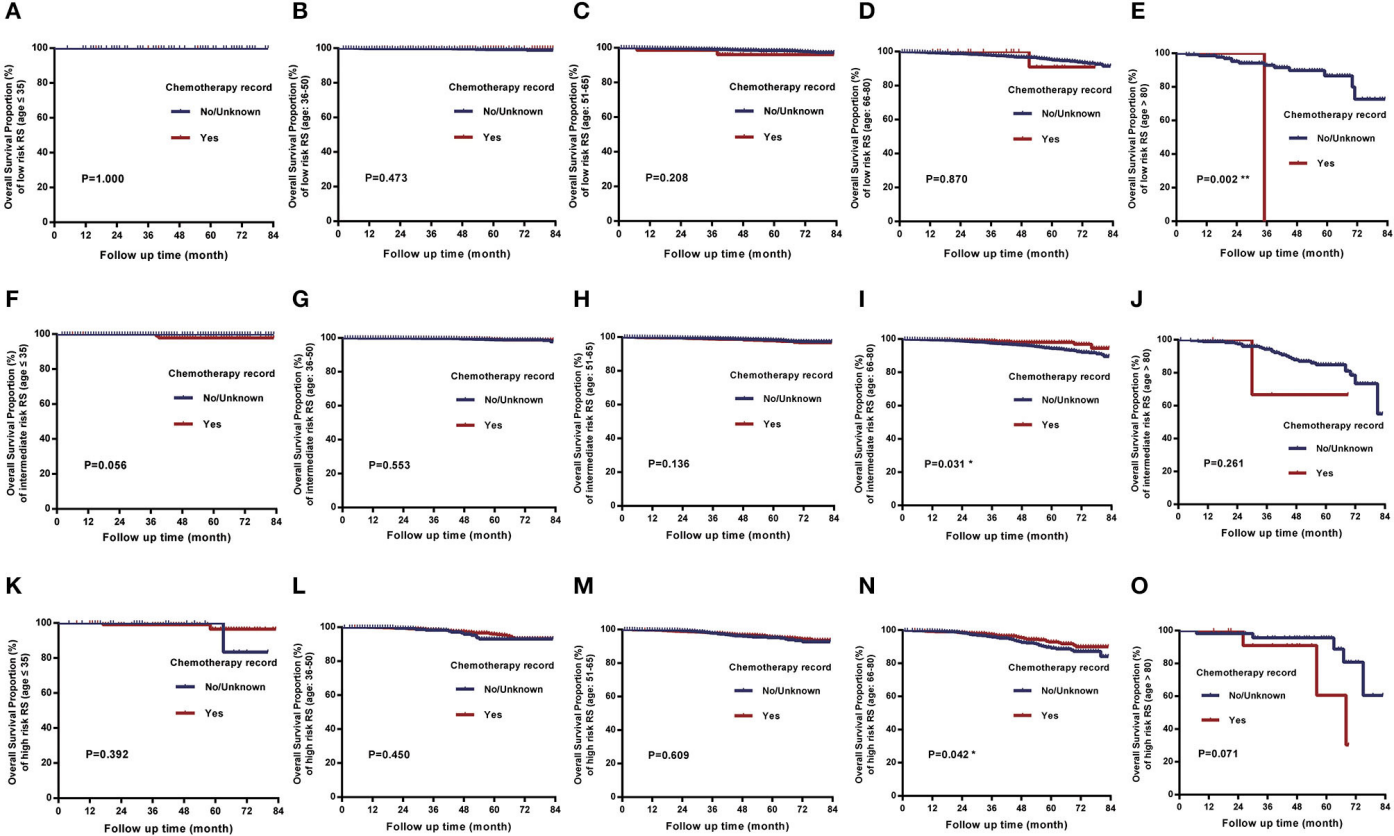
Comparison of overall survival between patients receiving chemotherapy and those not receiving chemotherapy or unknown in the five age groups. **(I, N)** There were significant differences of overall survival in age group of 66–80 years with intermediate-risk RS (*P* = 0.031) and high-risk RS (*P* = 0.042). **(E)** Women >80 years with low-risk RS had worse overall survival when receiving chemotherapy than those not or unknown (*P* = 0.002). **(J, O)** Women aged >80 years with intermediate-risk and high-risk RS, chemotherapy also seemed to be harmful although the differences were not statistically significant (*P* > 0.05). **(A–D, F–H, K–M)** There were no significant difference in any other age groups (*P* > 0.05).

In the comparison of overall survival between patients receiving chemotherapy and those not/unknown, we found that there were significant differences between two treatment groups when RS of 11–25 (*P* = 0.005) and RS of 26–100 (*P* = 0.006), while there was no significant difference when RS of 0–10 (*P* = 0.871) (Figure 4). Analysis of each age group separately revealed that for intermediate-risk and high-risk RS patients, there were significant differences in survival only in groups aged 66–80 years (*P* < 0.05), and the differences were not significant in the other age groups (all *P* > 0.05). Particularly noteworthy, women aged >80 years with low-risk RS had worse prognoses when they received chemotherapy than those not or unknown (*P* = 0.002) and women aged >80 years with intermediate-risk and high-risk RS, chemotherapy also seemed to be harmful although the differences were not statistically significant (Figure 5). In other words, when the overall survival as the evaluation criteria, the chemotherapy benefits in intermediate-risk and high-risk RS patients were only found in the 66–80 years of age groups, and chemotherapy did more harm than good to patients aged over 80 years. There were no significant differences between receiving chemotherapy and those not/unknown in all other age and risk groups.

## Discussion

In this study, we demonstrated that the distribution of Oncotype RS differ among various age groups of patients with HR+/HER2–, node-negative breast cancer. RS values tend to be higher in younger patients and lower in older patients. The percentage of patients with low-risk RS (0–10) increases with age, whereas that of patients with intermediate-risk RS (11–25) decreases with age (except for the group of 36–50 years, which had the highest rate of intermediate-risk RS). We also found that the proportion of patients receiving chemotherapy was related to the results of the Oncotype RS assay, patients at high risk RS category (26–100) being more likely to be treated with chemotherapy. Age stratification showed that the proportion of patients receiving chemotherapy was highest in the ≤35 years of age group, whereas it was lowest in the over 80 years of age group. The rates of receiving chemotherapy decreased with age in all risk categories. Patients aged ≤35 years with high-risk RS had the highest rate of receiving chemotherapy (82.0%), whereas patients aged >80 years with low-risk RS had the lowest rate (0.7%). We also compared the overall survivals between patients receiving chemotherapy and those not/unknown in each age group. Our findings could supplement those of current studies on the correlation between RS and administration of adjuvant chemotherapy, helping clinicians to decide which patients are most likely to benefit from receiving chemotherapy.

Recently, Zhang et al. examined data from 17 SEER registries concerning administration of chemotherapy and survival benefit of Oncotype RS in hormone receptor-positive breast cancer patients between 2004 and 2015. They reported that the usage of chemotherapy decreased significantly with low and intermediate RS, and increased for high RS among node-negative patients. Administering chemotherapy has been optimized on the basis of Oncotype DX results among HR+/N0 breast patients, leading them to better survivals than those not receiving Oncotype DX test (22). The authors accomplished a meaningful job, and they described the trend of reported chemotherapy by RS and the survival differences associated with Oncotype DX usage. However, they did not analyze the relationship between RS and chemotherapy benefit in certain sub-groups, such as age ranges. Poorvu et al. identified that patients aged <40 years with stage I–III HR+/HER2–/N0 breast cancer, a median follow-up of 6 years, RS ≥26 experienced substantial risk of early distant recurrence (23). Liu et al. used the SEER database to analyze the chemotherapy decision-making and prognosis for stage I and II, estrogen receptor-positive, node-negative breast cancer aged under 40 years of age, and found that such patients with intermediate-risk RS were significantly less likely to receive chemotherapy over time, whereas the percentages receiving chemotherapy were stable in the low-risk and high-risk RS cohorts. Being at high-risk RS, but not intermediate-risk RS, was associated with better breast cancer-specific survival when adjuvant chemotherapy was administered (24).

In the present study, we divided patients into five age groups and found that a greater proportion of women aged ≤35 years received chemotherapy than did women in any other age group, whereas those aged over 80 years were least likely to receive chemotherapy. However, in patients with intermediate- and high-risk RS, chemotherapy was associated with significant overall survival benefit only in patients aged 66–80 years, there were no significant overall survival benefits in intermediate- and high-risk members of any other age groups. Considering the adverse effects of chemotherapy on other important functions such as reproduction, whether all patients with RS ≥26 need to receive chemotherapy needs to be re-examined, especially in younger women. Furthermore, we found that chemotherapy seems to do more harm than good in eldest age group (>80 years) at all RS risk, further suggesting that administration of chemotherapy should be considered carefully.

In a retrospective review, Reyes et al. investigated the usage of chemotherapy in patients at intermediate-risk RS (11–25) to determine the potential for changes in practice based on TAILORx results (25). They found that younger patients (age ≤ 50 years) at lower-intermediate risk RS (11–15) were more likely to receive chemotherapy if they were treated at community or comprehensive centers, whereas moderate grade was also a significant factor in administration of chemotherapy to patients aged ≤50 years with an RS of 16–25. Other significant factors in older patients (age >50 years) with a RS of 11–25 including black race, estrogen receptor-positive/progesterone receptor-negative (ER+/PR–), and moderate/high grade. Thus, there are still no precise guidelines for administration of adjuvant chemotherapy to breast cancer patients with intermediate-risk RS, meaning that doctors may play a considerable role in making these decisions.

Overall survivals were compared in the present study, while the main consideration in TAILORx were the recurrence of disease (9, 12). This partly explains why the survival differences were not significant in many age groups even at a high-risk RS category. However, for cancer patients, they were probably more concerned with how long she has left to live, so it would make sense to compare the overall survivals. Table 2 summarizes and compares the cohort characteristics and results regarding age stratification in the present study and TAILORx.

**Table 2:** Summary table comparing cohort characteristics and results regarding age stratification in the present study and TAILORx.

	**The recent TAILORx [1; 2; 3]**	**The present study**
Research category	Prospective trial	Retrospective research
Main analysis set	9,719 eligible patients with follow–up information	49,539 eligible patients with follow–up information
Clinicopathological feature	HR+/ HER2–/ N0	HR+/ HER2–/ N0
Registration time	April 2006 – October 2010	January 2010 – December 2015
RS distribution	0–10 17% (1619/9719)	0–10 22.5% (11164/49539)
	11–25 69% (6711/9719)	11–25 64.1% (31731/49539)
	26–100 14% (1389/9719)	26–100 13.4% (6644/49539)
Summary of the results regarding to age stratification	A low proportion of distant recurrence at 9 years with endocrine therapy alone if the RS was 0–15, irrespective of age	The mean RS were different among five age groups (≤35, 36–50, 51–65, 66–80, and > 80 years of age)
	Age > 50 with a RS of 0–25, and ≤ 50 with a RS of 0–15, endocrine therapy was non-inferior to chemoendocrine therapy	The most common age group was 51–65 years, followed by 36–50 years, 66–80 years, ≤35 years, and > 80 years
	Age ≤ 50, chemotherapy was associated with some benefit for women who had a RS of 16–25	The percentage of low–risk RS (0–10) patients increased with age
	Age ≤ 50 with high clinical risk and RS (11–25) who received endocrine therapy alone, and those RS (26–100) who received chemoendocrine therapy, the distant recurrence rate at 9 years exceeded 10%	The percentage of intermediate–risk RS (11–25) patients decreased with age except for the group of 36–50 years, which has the highest rate of intermediate risk RS
	Age ≤ 50 and RS (11–25), endocrine therapy was noninferior to chemoendocrine therapy at 9 years if clinical risk was low; while chemotherapy was associated with benefit if clinical risk was high	The group aged ≤ 35 years has the greatest rate of high–risk RS
	Age > 50, endocrine therapy was noninferior to chemoendocrine therapy in the cohort with a RS of 11–25, regardless of clinical risk category	The proportion of receiving chemotherapy decreases with age in all RS risk categories
	Age ≤ 50, distant recurrence rate at 9 years were very low among women with a RS of 0–10, irrespective of clinical–risk category	Age ≤ 35 with RS of 26–100 had the highest chemotherapy receipt rate, while age > 80 with RS of 0–10 had the lowest chemotherapy receipt rate
	The chemotherapy benefit was most evident at 45 years of age in premenopausal women and waned at younger and older ages and with menopause	Overall survival was benefited by chemotherapy only in the age group of 66–80 years of age with intermediate- and high-risk RS
	There were significant interactions between chemotherapy treatment and age (≤50 vs. 51 to 65 vs. >65 years) for invasive disease–free survival (*P* = 0.03) and for freedom from recurrence of breast cancer at a distant or local–regional site (*P* = 0.02) but not at a distant site (*P* = 0.12)	Age > 80, the chemotherapy seemed to do more harm than good
		

The present study has numerous strengths. Most importantly, it was an extensive, population-based retrospective study using SEER cancer registries, which include more than 95% of patients with cancer within geographically dispersed catchment areas that cover ~28% of the population of the USA (26, 27). Therefore, our results are representative of breast cancer in the USA and estimates on small subgroups are robust. What is more, we not only analyzed the RS distribution and chemotherapy status in different age groups, but also explored the relationship between RS and chemotherapy benefit according to the detailed age grouping. These findings can be provided to clinical researchers as a reference for conducting clinical trials and can also help in making individualized clinical decisions.

There are some limitations that must be considered when interpreting the results of the present study. The chemotherapy information recorded in the SEER database includes only “Yes” and “No/unknown.” Thus, some patients who were recorded as “No/unknown” may have received chemotherapy. However, it is not possible to obtain more accurate information from the SEER database concerning these patients, this is therefore the main limitation of this study. Our findings could shed new light on the effect of age on Oncotype DX Breast Recurrence Scores and chemotherapy use, however, further validation is needed for this virtual study. In the future, we plan to not only validate the results on our own data, but also develop a prediction model for whether a breast cancer patient with a certain Oncotype DX RS and of a certain age should be given chemotherapy or not. Meanwhile, some other potential limitations and biases of the SEER database should also be taken into consideration (28, 29).

In summary, in the present study we identified the distribution of RS, the proportion of receiving chemotherapy, and the relationship between RS and chemotherapy benefit according to a detailed age grouping for women with HR-positive, HER2-negative, and node-negative breast cancer, which may help in making individualized clinical decisions.

## Data Availability Statement

Publicly available datasets were analyzed in this study. This data can be found at: https://seer.cancer.gov/.

## Author Contributions

JW conceived and designed the idea to this paper. RC and XK collected the data. RC analyzed the data and drafted the paper. XK and XW analyzed the data. YF revised the final paper. All authors read and approved the final manuscript.

## Conflict of Interest

The authors declare that the research was conducted in the absence of any commercial or financial relationships that could be construed as a potential conflict of interest.
